# Combination of anginex gene therapy and radiation decelerates the growth and pulmonary metastasis of human osteosarcoma xenografts

**DOI:** 10.1002/cam4.1476

**Published:** 2018-04-16

**Authors:** Kai Zhao, Shang‐You Yang, Jun Geng, Xuan Gong, Weiming Gong, Lin Shen, Bin Ning

**Affiliations:** ^1^ Jinan Central Hospital Affiliated to Shandong University No. 105, Jiefang Road Jinan 250013 Shandong China; ^2^ Department of Surgery, Orthopedics University of Kansas School of Medicine‐Wichita Wichita 67214 Kansas

**Keywords:** Anginex, anti‐angiogenesis agent, gene therapy, osteosarcoma, pulmonary metastasis, radiotherapy

## Abstract

Investigate whether rAAV‐anginex gene therapy combined with radiotherapy could decrease growth and pulmonary metastasis of osteosarcoma in mice and examine the mechanisms involved in this therapeutic strategy. During in vitro experiment, multiple treatment regimes (rAAV‐eGFP, radiotherapy, rAAV‐anginex, combination therapy) were applied to determine effects on proliferation of endothelial cells (ECs) and G‐292 osteosarcoma cells. During in vivo analysis, the same multiple treatment regimes were applied to osteosarcoma tumor‐bearing mice. Use microcomputed tomography to evaluate tumor size. Eight weeks after tumor cell inoculation, immunohistochemistry was used to assess the therapeutic efficacy according to microvessel density (MVD), proliferating cell nuclear antigen (PCNA), and terminal‐deoxynucleotidyl transferase‐mediated nick‐end labeling (TUNEL) assays. Metastasis of lungs was also evaluated by measuring number of metastatic nodules and wet weight of metastases. The proliferation of ECs and the tumor volumes in combination therapy group were inhibited more effectively than the other three groups at end point (*P* < 0.05). Cell clone assay showed anginex had radiosensitization effect on ECs. Immunohistochemistry showed tumors from mice treated with combination therapy exhibited the lowest MVD and proliferation rate, with highest apoptosis rate, as confirmed by IHC staining for CD34 and PCNA and TUNEL assays (*P* < 0.05). Combination therapy also induced the fewest metastatic nodules and lowest wet weights of the lungs (*P* < 0.05). rAAV‐anginex combined with radiotherapy induced apoptosis of osteosarcoma cells and inhibited tumor growth and pulmonary metastasis on the experimental osteosarcoma models. We conclude that the primary mechanism of this process may be due to sensitizing effect of anginex to radiotherapy.

## Introduction

Osteosarcoma is the most common primary malignant tumor of the bone and has a high incidence in children and adolescents. Because of its invasive growth and early pulmonary metastasis, osteosarcoma often results in poor prognosis 1. In the past two decades, the routine management of osteosarcoma has involved a combination of chemotherapy and radical surgical resection; this approach has increased the 5‐year survival rate from 50% to 70% in patients who do not have metastatic diseases at presentation 2, 3, 4. However, for patients with early metastasis, those who react poorly to chemotherapy, and those who develop recurrent disease after surgical treatment, the prognosis remains poor. Additionally, considering the long‐term and short‐term toxicities of chemotherapeutic agents commonly used to treat osteosarcoma, alternative treatments are required to support conventional management strategies and improve survival rates in patients with this malignancy.

Novel anti‐angiogenic therapies may represent an effective approach for the treatment of osteosarcoma 5, 6; these therapies aim to suppress or eradicate tumors by interfering with blood supply to the tumor. Anginex is one such artificial *β*‐sheet‐forming peptide with potent anti‐angiogenic activity. Anginex was designed based on the sequence and three‐dimensional structure of other anti‐angiogenic agents, including platelet factor‐4, interleukin‐8, and bactericidal permeability‐increasing protein (BPI) 7. Moreover, anginex, which has been shown to efficiently block endothelial cell (EC) growth, acts by specifically blocking the adhesion and migration of angiogenically activated ECs, leading to apoptosis and ultimately to inhibition of angiogenesis in vitro and in vivo 8. The antitumor activity of anginex has been demonstrated in many previous studies 7, 8, 9, 10, although few studies have focused on its therapeutic effects in osteosarcoma. Previous studies have shown that anginex alone is not as effective as when combined with radiotherapy or chemotherapy 11, 12. Although the mechanism is still controversial, some researchers have proposed the “vascular normalization theory” 13, in which more efficient delivery of drugs and oxygen to the targeted cancer cells is achieved through the modified vessels following treatment with anti‐angiogenic agents 14. This would make the cancer cells more sensitive to radiotherapy and chemotherapy, thereby increasing the efficacy of treatment. However, others have proposed that anginex has a radiosensitizing effect on the ECs, in which ECs would be more sensitive to anginex after radiotherapy, resulting in increased induction of apoptosis 12.

Therefore, in this study, we evaluated the inhibitory effects of anginex combined with radiotherapy on the growth and pulmonary metastasis of osteosarcoma, which is not sensitive to radiation. Because of the short half‐life and low biological availability of the peptide itself, we chose to use the recombinant adeno‐associated virus (rAAV)‐anginex‐enhanced green fluorescent protein (eGFP) construct, a rAAV carrying cDNA of anginex and eGFP 10 that possessed comparable angiostatic properties as the synthetic peptide 15. The in vitro anti‐angiogenic effects of this peptide have been studied extensively in previous studies, and its anti‐angiogenic mechanisms were also revealed 16. This study investigated the inhibitory effects of anginex on an in vivo osteosarcoma model using severe combined immunodeficient (SCID) mice and the effects of a combination therapy with radiotherapy. We used the classic SCID model of osteosarcoma 17, in which SCID mice act as hosts for inoculation of G‐292 human osteosarcoma cells. Our results demonstrated that anginex combined therapy exhibited strong inhibition of tumor blood vessels and the growth and metastasis of the tumor itself, making it a promising agent with potential applications in the treatment of osteosarcoma.

## Materials and Methods

### Materials and antibodies

The rAAV vectors encoding anginex and eGFP (titer: 1 × 10^10 ^vg/mL) were kindly provided by Dr. DanFeng Dong of Xi'an Jiaotong University. The human osteosarcoma cell line G‐292 and human umbilical vein endothelial cells (HUVECs) were both purchased from American Type Culture Collection (Manassas, VA). McCoy's 5a medium, RPMI 1640, phosphate‐buffered saline (PBS), fetal bovine serum (FBS), and 0.25% trypsin were purchased from Gibco BRL (Life Technologies, Inc, Rockville, MD). 3‐(4,5‐Dimethylthiazol‐2‐yl)‐2,5‐diphenyltetrazolium bromide (MTT) reagent was purchased from BD Biosciences (Bedford, MA). Anti‐CD34 antibodies, antiproliferating cell nuclear antigen (PCNA) antibodies, and the terminal‐deoxynucleotidyl transferase‐mediated nick‐end labeling (TUNEL) kit were commercially available in Santa Cruz Biotechnology (Santa Cruz, CA).

### Cell culture and conditions

The human osteosarcoma cells (G‐292) were cultured in McCoy's 5a medium supplemented with 10% FBS and 1% penicillin/streptomycin at 37°C in a humidified 5% CO_2_ incubator. Cells were subcultured at 1:3 twice a week when they reached 75–80% confluence. HUVECs were cultured in the same manner, except that RPMI 1640 medium was used, and cells were passaged at a 1:2 ratio twice a week.

### Animals

Thirty SCID mice (4 weeks of age) were purchased germ‐free from Shanghai SLAC Laboratory Animal Co. Ltd. (Shanghai, China). Mice were allowed to acclimate for 1 week before inoculation and were housed in sterile cages at the Orthopaedic Department Sterile Facility with an artificial 12‐h light/12‐h dark cycle. Mice were fed irradiated rat chow and acidified (pH 2.5) tap water. Before manipulations, including tumor inoculation and euthanasia, mice were anesthetized with an intraperitoneal injection of 50 mg/kg ketamine, 5 mg/kg xylazine, and 0.75 mg/kg acepromazine.

### In vitro study

#### rAAV‐anginex gene transfection

G‐292 cells were seeded in 96‐well plates at a density of 5000 cells/well with McCoy's 5a medium complemented with 10% FBS. After 12 h, the medium was changed into FBS‐free McCoy's 5a medium supplemented with 100 *μ*L of 10^10 ^vg/mL rAAV‐anginex, yielding a multiplicity of infection (MOI) of 10^5^. Four hours later, the G‐292 cells were washed with PBS twice, followed by addition of 200 *μ*L of full McCoy's 5a medium to each well. Forty‐eight hours after viral vector transduction, images of G‐292 cells were acquired using a fluorescence microscope, and the efficiency of transgene production was calculated, and also the morphology of G‐292 cells was observed under light microscope.

#### Analysis of HUVEC proliferation using MTT colorimetric assays

To investigate the inhibitive effect of radiotherapy and rAAV‐anginex on the HUVECs and G‐292 cells, the proliferation of the two cell lines was separately measured by MTT assay. The two cells were separately seeded in 96‐well plates and randomly divided into four groups for each cell line: (1) rAAV‐eGFP control group (control group), in which each well was treated with 100 *μ*L of 10^10 ^vg/mL rAAV‐eGFP; (2) radiotherapy group, in which 24 h after cell seeding, cells were irradiated at 2 Gy; (3) rAAV‐anginex treatment group (anginex group), in which each well was treated with 100 *μ*L of 10^10 ^vg/mL rAAV‐anginex‐eGFP; (4) combination treatment group (combination group), in which cells were treated with 100 *μ*L of 10^10 ^vg/mL rAAV‐anginex and then irradiated with 2 Gy at 24 h after anginex treatment. Cells were then incubated for up to 72 h at 37°C in an atmosphere containing 5% CO_2_; 50 *μ*L of 5 mg/mL MTT was added to wells at 12, 24, 36, 48, 60, and 72 h. Absorbance was read at 570 nm using a DIAS Microplate Reader. The proliferation values were recorded as the absorbance values.

#### Test of the radiosensitizing effects of anginex on ECs using plate clonogenic formation assay

ECs and G‐292 cells were separately seeded in 6‐well plate with 100, 200, 400, 600, 800, 1000 cells in one well, and 24 h later when cells attached to the wall, both of cells were randomly divided into the following groups, respectively: (1) radiotherapy only group (control group): wells with different gradient cells were successively irradiated at 0, 2, 4, 6, 8, 10 Gy; (2) combination treatment group (combination group): this group was treated with 1 *μ*L of 10^10 ^vg/mL rAAV‐anginex, which made MOI at 10^5^ and then received radiation at 0, 2, 4, 6, 8, 10 Gy. After that, these cells were cultured at 37°C in 5% CO_2_ and saturated humidity environment for 2 weeks. Stop the assay once clones were visible to the naked eye. The colonies were stained with 0.1% Crystal violet (Sigma) for 1 h 37°C, and then, we counted clones which containing cells above 50 by microscope. Using the equation: plating efficiency (PE) = number of clones in control group/cells of plating × 100%; then used PF as correction factor to calculate surviving fraction (SF): SF = number of clones in combination group/(cells of plating×PF). Use the single‐hit multitarget (SHMT) model to calculate integrated cell survival curves,quasithreshold dose (Dq), mean lethal dose (D0), and survival fraction of 2 Gy (SF2). The radiosensitization effect was represented as radiosensitization rate (SER_D0_) = D0 of control group/D0 of combination group.

### In vivo study

#### Establishment of the osteosarcoma model

G‐292 cells at logarithmic growth phase were trypsinized, resuspended in FBS‐free RPMI 1640 medium to a concentration of 1 × 10^7 ^cells/mL, and then taken up into a 1‐mL syringe. All animal procedures were approved by the Institutional Animal Ethics Committee of Jinan Central Hospital Affiliated to Shandong University. After inducing anesthesia in the SCID mice, the needle of the syringe was inserted into the proximal part of the tibial tuberosity of the right leg with a drill‐like motion to prevent cortical fracture. Once the cortex was traversed, the needle was inserted into the metaphysis and diaphysis of the bone by injection of 10‐*μ*L suspensions (10^5^ cells) into the medullary cavity. After all inoculations were completed, radiographic examination was performed to determine whether injection led to cortex fracture.

#### Treatment variation

Radiographic examination was performed at the end of the second week after tumor inoculation, revealing a tumor inoculation percentage of 86.7%. Twenty‐four mice were randomly selected from mice showing successful tumor inoculation and then randomly divided into four groups: the rAAV‐eGFP control group (control group), the radiotherapy treatment group (radiotherapy group), the rAAV‐anginex treatment group (anginex group), and the combination treatment group (combination group), with six mice in each group.

The treatment was first given to each group through multisite intertumor injection when the tumor grew to a visible size at the third week after tumor inoculation. Animals in the control group received rAAV‐eGFP at 1 × 10^9 ^vg/tumor, animals in the radiotherapy group received local irradiation with 2 Gy once a week for 5 weeks, and animals in the anginex group received rAAV‐anginex at 1 × 10^9 ^vg/tumor. For the combination group, animals received 1 × 10^9 ^vg/tumor, and 4 days later, tumor‐bearing mice received local irradiation with 2 Gy once a week for 5 weeks.

All animals were monitored daily by visual inspection to evaluate general health and tumor development. All animals were sacrificed at 8 weeks after tumor cell inoculation.

#### Microcomputed tomography (CT) evaluation and tumor volume measurements

After inoculation, SCID mice were subjected to micro‐CT (GE Medical Systems, London, ON, Canada) examinations every week to monitor tumor growth. Scan parameters were as follows: isotropic voxel size, 45 *μ*m; projections, 400; exposure time, 400 msec; voltage, 80 kW; and current, 450 *μ*A. Tumor volume was determined by measuring the diameters of tumors in micro‐CT images and calculated by the equation *V* = 4/3π (1/4[D1 + D2])^2^, where D1 is the width of the tumor and D2 is the length of the tumor.

### Histological and morphometric analysis of the tibial osteosarcoma tissue and pulmonary metastasis nodules

The tumor tissues of the tibiae and lungs were harvested from mice, washed in PBS, fixed in PBS‐buffered 3.7% formalin. Paraffin‐embedded sectioning was performed and stained with hematoxylin and eosin (HE). The slides of tibiae were viewed and photographed under a fluorescence microscope to determine the transduction efficiency of rAAV‐eGFP.

Pulmonary metastasis was assessed by counting the number of nodules from a macroscopic view and using pathological slides with HE staining. The wet weight of the lungs was also measured.

### Immunohistochemistry (IHC) of paraffin‐embedded tissues

The tumor tissue from the tibia was sliced into 7‐*μ*m‐thick sections and embedded in paraffin. SP‐ABC assays were then used for IHC.

To assess the microvessel density (MVD), immunohistochemical staining was performed using anti‐CD34 antibodies (Santa Cruz Biotechnology), as detailed previously 5. To quantify tumor MVD, paraffin sections were fixed and stained with anti‐CD34 primary antibodies. ECs stained in brown and were CD34 positive. Single ECs and EC clusters stained as CD34 positive were counted as a single vessel. For each tumor, the sections were examined at a magnification of 100 ×  under a light microscope, three regions of highest MVD were chosen, and the microvessels in these regions were quantified at a magnification of 100×. The number of microvessels was then recorded. The average number was calculated as the MVD of the tumor. The branching and length of the vessels were also determined at a magnification of 100× under a light microscope in three different regions.

The proliferation and apoptosis of the primary tumor tissues were assessed by PCNA and TUNEL staining (Santa Cruz Biotechnology), respectively. To quantify PCNA and TUNEL staining, the number of positive cells and total number of tumor cells were counted in five random fields at 100× magnification, followed by calculation of the percentage of positive cells.

All IHC examinations were individually evaluated by two different experienced pathologists.

### Statistical analysis

Statistical analysis of differences between groups was performed using ANOVA (Analysis of Variance) with SPSS 18.0 software (for Windows). Differences with *P* values of <0.05 were considered significant. Data are expressed as means ± standard deviations.

## Result

### Combination treatment inhibited the proliferation of HUVECs in vitro

**Figure 1:**
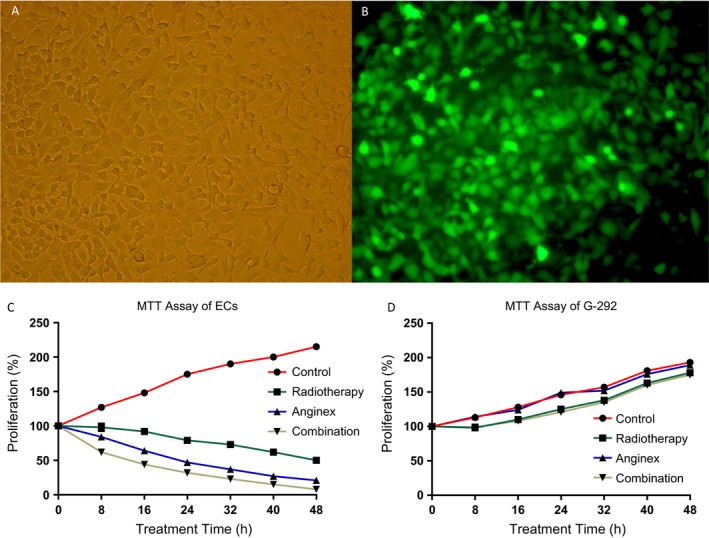
(A) The cellular morphology of G‐292 under light microscope, and B was the cellular morphology of G‐292 of the same culture plate under fluorescence microscope. When MOI at 105, the transfection efficiency of the human osteosarcoma cell line G‐292 was 85.78% (A, B). Anginex inhibited proliferation of ECs in vitro. Every 8h, MTT assay was performed in the separate four groups of endothelial cells and G‐292 cells (control group, treated with rAAV‐eGFP; radiotherapy group: 24 h after cell seeding, radiation at 2 Gy was used; anginex group: each well was treated with 100 *μ*L of 10^10 ^vg/mL rAAV‐anginex; combination treatment group: this group was treated with 100 *μ*L of 10^10 ^vg/mL rAAV‐anginex, and 24 h later receive radiation at 2 Gy). The absorbance of cell lysates at 570 nm was read. The results were expressed as mean proliferation rate ± SD. C shows: in both anginex group and combination group, the proliferation of Ecs was inhibited significantly, there was also significant difference between the two groups; D shows the radiotherapy and the combination treatment were both effective at inhibiting the proliferation of G‐292 cell line, but the difference of the two groups with the other two was small.

Forty‐eight hours after transfection, images of G‐292 cells were acquired under a fluorescence microscope to evaluate the transgene production of rAAV‐anginex‐eGFP. As shown in Figure 1A and B, when the MOI was 10^5^, the transduction efficiency of the G‐292 cells was 85.78%, and under the light microscope, we saw that the gene transfer did not substantially affect the morphology of G‐292 cells (Fig. 1A and B).

The MTT results of ECs and G‐292 cell lines demonstrated that ECs in the combination, radiotherapy, and anginex groups exhibited significant decreases in proliferation capacity compared to ECs in control groups (*P *<* *0.05), and the proliferation capacity of ECs in the combination group was significantly weak compared with that in the anginex group (*P *<* *0.05) (Fig. 1C). In contrast, for G‐292 cells, the radiotherapy and combination groups showed the same tendency toward inhibition of proliferation, suggesting that the inhibitive effects were mostly related to radiation, not anginex. But compared with the significant inhibitive result of Ecs by radiotherapy, the effect of radiation on G‐292 cells was very limited, most because this osteosarcoma cell was not very sensitive to radiotherapy, at least under this radiation equivalent (2 Gy) (Fig. 1D).

### Anginex had radiosensitizing effect on ECs

**Figure 2:**
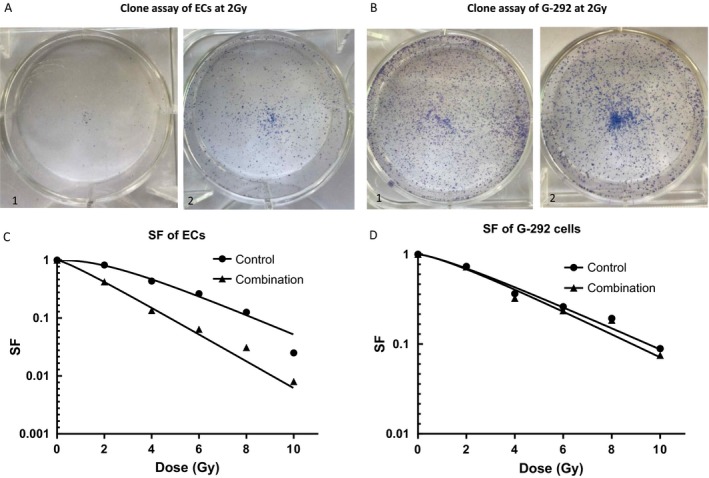
(A, B) The images of cells clone assay of ECs and G‐292 cells at the radiation dose of 2 Gy. A‐1 is the control group of ECs, which had more cell clones than the combination group (A‐2), while B is the clone images of G‐292 cells at the radiation dose of 2 Gy, which had the no significant difference between the two groups (B1‐2). (C, D) The integrated cell survival curves of the two cell lines. In the ECs, as the radiation dose rose, the SF difference between the control group and the combination group was becoming significant, while the trend of G‐292 cells was nearly the same.

At the endpoint of the clone formation assay, the ECs and G‐292 cells showed different trends. ECs of combination group had fewer clones than the control group (Fig. 2A1‐2), while the G‐292 cells of both groups had the same clone morphology (Fig. 2B1‐2).

Integrated cell survival curves of the two cells were shown in Figure 2C and D, and the Dq, D0, SF2 of the ECs in combination group were respectively 2.43, 1.88, and 0.42, which was significantly lower than the control group (6.96, 2.56, 0.82), while the same parameters of G‐292 cells has no significant difference (Table 1). SER_D0_ of Ecs was 1.36, while the SER_D0_ of G‐292 cells was 1.05, which means anginex had radiosensitizing on ECs rather than G‐292 cells.

**Table 1:** The Dq, D0, SF2, and SER_D0_ of the ECs group and the G‐292 cell line group

Groups	Dq	D0	SF2	SER_D0_
Control (ECs)	6.96	2.56	0.82	1.36
Combination (ECs)	2.43	1.88	0.42	
Control (G‐292)	5.04	3.62	0.74	1.05
Combination (G‐292)	4.95	3.45	0.72	

In the ECs, the Dq, D0, SF2, and SER_D0_ of the combination group were 2.43, 1.88, and 0.42, which was significantly different from the control group, which was 6.96, 2.56, and 0.82. While in the G‐292 cell line, the three parameters were nearly the same.

### Suppression of tumor growth by intratumoral injection of the rAAV‐anginex vector combined with radiotherapy

**Figure 3:**
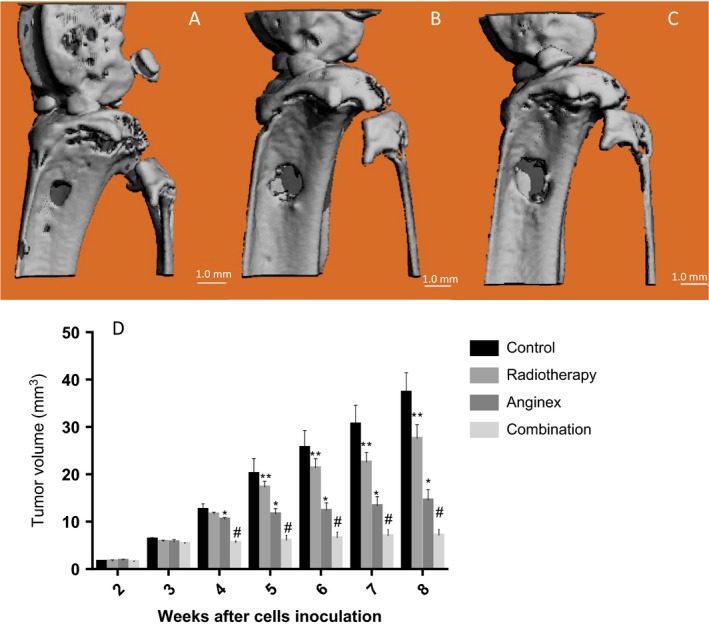
At the second week after tumor cells inoculation, the typical primary osteosarcoma was seen from micro‐CT scan (A). The tumor was located in the tubercle of tibia with cortical destructions and periosteal reaction, and as time passed by, the lesion became bigger (B, C). Tumor volumes were calculated using the equation *V* = 4/3π[1/4(D1 + D2)]2, where D1 is the width and D2 is the length of the tumor scanned from micro‐CT. The size of Orthotopic tumor since the second week after inoculation of the G‐292 cells was seen in D. Since the fourth week, anginex group showed decelerated growth of tumor, while from the fifth week, tumor of the combination group grew slower than the other three groups and maintained the same tendency until the end point. Data are presented as mean volume ± SD (*n* = 6, Bars represent average value. Error bars represent standard deviations.*means compare with control and radiotherapy group *P* < 0.05, #means compare with the other three groups, *P* < 0.05).

After transplantation of the tumor cells, orthotopic osteosarcoma was observed by micro‐CT in the proximal tibiae from the third week after tumor cell inoculation. The tibial tumors were then injected directly with the rAAV‐eGFP or rAAV‐anginex vector. In the radiotherapy group, mice were irradiated with 2 Gy at the lesion area for 5 weeks, and in the combination group, radiation at the same dose was administered at the lesion area 4 days after injection of rAAV‐anginex. Every week, micro‐CT scans were used to monitor the development and growth of the primary tumor (Fig. 3A–C).

Using the MicroView program, we found that tumor volumes at week 8 after tumor cell inoculation were 37.25 ± 4.23 mm^3^ in the control group, 27.50 ± 3.02 mm^3^ in the radiotherapy group, 14.51 ± 2.25 mm^3^ in the anginex group, and 7.12 ± 1.24 mm^3^ in the combination group; these differences were statistically significant (Fig. 3D). Anginex treatment strongly inhibited tumor growth, but the combination treatment showed significantly synergetic effects compared to anginex or radiation alone (*P *<* *0.05). Also, the radiotherapy alone could decrease the tumor volume, as the tumor cell is not so sensitive to radiation at this radiation equivalent. Therefore, we concluded that the decrease in tumor volume in combination group was due to the effect of anginex and radiotherapy on ECs rather than the tumor cell itself.

**Figure 4:**
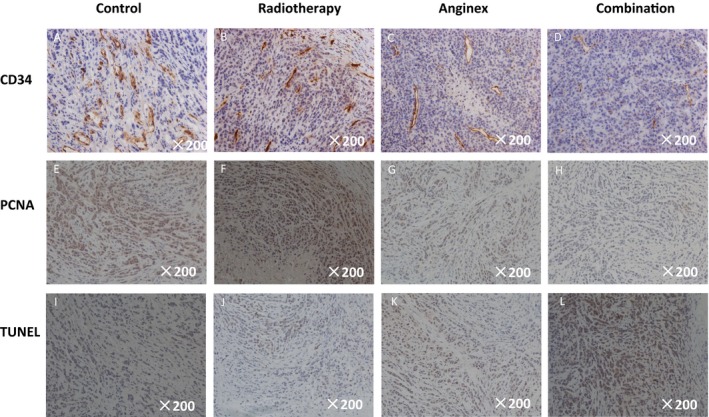
The ICH of the tumor slides at the eighth week after tumor cell inoculation was shown in figure (×200). The CD 34 staining of the primary tibial osteosarcoma in the different groups was shown in A–D, which reflects the density of microvessels. The MVD of anginex and combination group was smaller than the other two groups, while the combination group had the lowest MVD. The PCNA staining of the primary tibial osteosarcoma in the different groups was shown in E–H, which reflected the proliferation of the tumor. The yellow‐stained nucleus which represented the proliferation of tumor cells was obviously seen in control and radiotherapy groups, moderately seen in anginex group, but seldom seen in combination group. The Tunel staining of the primary tibial osteosarcoma in the different groups was shown in I–L, which reflected the apoptosis of the tumor. The yellow‐stained nucleus which represented the apoptosis of tumor cells was obviously seen in combination group, moderately seen in anginex group, but seldom seen in control and radiotherapy groups.

**Figure 5:**
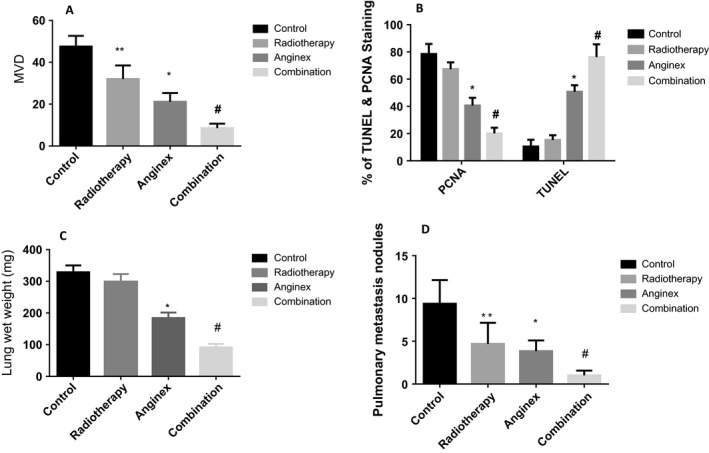
The column chart of MVD, PCNA, Tunel, lung wet weight, and the pulmonary metastasis nodules was shown in figure. The MVD of the orthotopic tumor of each group at the eighth week was shown in A. Compared with the control and radiotherapy groups, anginex and combination group had the lower MVD, but there was difference between them, which meant the combination group had the lowest MVD. The PCNA and Tunel of the orthotopic tumor of each group at the eighth week was shown in B. Compared with the control and radiotherapy groups, anginex and combination group had the lower proliferation rate and higher apoptosis rate, but there was also difference between them. The lung wet weight and pulmonary metastasis nodules of each group at the eighth week were shown in C. Compared with the other three groups, the combination group had the lowest lung weight and the fewest metastasis nodules, which was shown in D (*n* = 6, Bars represent average value. Error bars represent standard deviations. **means compare with control group *P* < 0.05, *means compare with control and radiotherapy group *P* < 0.05, #means compare with the other three groups, *P* < 0.05).

Immunohistochemical staining of the tumors with anti‐CD34 antibodies on week 8 after osteosarcoma implantation showed that the combination treatment significantly reduced the number of blood vessels in the tumors when compared with those in the other groups. On week 8 after tumor formation, the blood vessel counts were 47.5 ± 7.55 in the control group, 31.7 ± 5.23 in the radiotherapy group, 21.66 ± 4.55 in the anginex group, and 14.35 ± 2.35 in the combination group (Figs 4A–D and 5A); the intergroup difference was significant (*P *<* *0.05). And the combination treatment effectively induced apoptosis in ECs, making it difficult to count the vessels.

Additionally, PCNA and TUNEL staining in the combination group were significantly different from those in the other three groups. The proliferation index in the combination treatment group was lower than those in the other groups by PCNA staining and higher than those in the other three groups by TUNEL staining (PCNA: 20.15% ± 4.12%; TUNEL: 76.21% ± 9.41%; Figs 4E–L and 5B).

### Inhibitory effects of combination treatment on pulmonary metastasis

At the eighth week after tumor inoculation, mice in the combination groups resulted in a marked less metastatic nodules in the lungs in comparison with the groups (Fig. 5C and D). In addition, the combination group had the lowest lung wet weight (*P *<* *0.05). In contrast, two mice in the control group developed huge metastatic lesions that the nodular capsular surface even adhered to the pericardium.

All the data including MVD, branch points, vessel length, PCNA (%), TUNEL (%), lung wet weight (mg) pulmonary metastasis nodules of the four groups are detailed in Table 2.

**Table 2:** The MVD, branch points, vessel length, PCNA, Tunel, lung wet weight, and pulmonary nodules

	MVD	Branch points	Vessel length	PCNA (%)	TUNEL (%)	Lung wet weight (mg)	Pulmonary nodules
Control	10,027.21 ± 2014.65	9.44 ± 1.48	12.65 ± 1.69	78.46 ± 7.45	10.49 ± 4.98	328.45 ± 21.57	9.38 ± 2.76
Radiotherapy	9258.56 ± 1835.42^a^	8.68 ± 1.36	11.36 ± 1.97	67.35 ± 5.01	15.25 ± 3.54	298.21 ± 24.68	4.698 ± 2.46^a^
Anginex	5682.25 ± 1536.58^b^	3.14 ± 0.87^b^	9.64 ± 1.35^b^	40.61 ± 5.66^b^	50.69 ± 4.89^b^	184.15 ± 17.25^b^	3.85 ± 1.24^b^
Combination	3616.68 ± 1265.68^c^	1.35 ± 0.54^c^	6.25 ± 1.06^c^	20.15 ± 4.12^c^	76.21 ± 9.41^c^	91.54 ± 10.88^c^	1.02 ± 0.55^c^

*n* = 6, Bars represent average value. Error bars represent standard deviations.

a Means compare with control group *P* < 0.05

b Means compare with control and radiotherapy group *P* < 0.05

c Means compare with the other three groups, *P* < 0.05.

## Discussion

Since the discovery that the development, growth, spread, and invasion of malignant tumors depend on angiogenesis, researchers have focused on the potential clinical treatment strategies targeting angiogenesis. Angiogenesis plays an important role in the development, growth, metastasis, and recurrence of tumors, including osteosarcoma, and provides oxygen and nutrients to the tumor cells 18. Importantly, anti‐angiogenic agents can arrest tumor growth and prevent metastasis formation because tumors generally cannot grow beyond 1–2 mm in diameter without formation of new blood vessels to supply nutrients and oxygen and to remove waste products 19, 20. ECs, which are involved in the processes of angiogenesis, can act as the therapeutic target because ECs are more accessible than other cells to pharmacologic agents delivered via the blood. Additionally, ECs are genetically stable and are not easily mutated into drug‐resistant variants 7, 8.

Some anti‐angiogenic agents such as endostatin 21, vasostatin 22, thrombospondin 23, angiostatin 24, and BPI 25 have been successfully produced to inhibit the growth and adhesion of ECs, thus resulting in tumor cell apoptosis. Many types of cancer, including osteosarcoma, have been shown sensitive to these anti‐angiogenic agents 5, 6, 26. However, there are still challenges preventing the wide applications of these agents, including drug resistance, renal and hepatic toxicity, and inhibition of normal vascularization. The development of the artificially synthesized peptide anginex helped to overcome some of these problems. Studies have revealed that anginex acts by specifically blocking the adhesion and migration of angiogenically activated ECs, leading to apoptosis and ultimately to inhibition of angiogenesis in vitro and in vivo 8, with minor toxicity to normal tissues and organs 18. Previous studies have also demonstrated the inhibitory effects of this drug on the growth and metastasis of some types of tumors, including human ovarian carcinoma 12, human hepatoma 10, and other cancers 18. However, the effects of anginex on osteosarcoma are still unknown. In this study, we used rAAV‐mediated gene transfer technique to investigate the inhibitory effects of anginex in osteosarcoma.

Compared with retroviruses and adenoviruses, AAV appears more efficient, less host inflammatory response, and longer expression period following in vivo transduction 27. These features make AAV an ideal carrier in gene therapy applications. Moreover, AAV is a nonpathogenic, replication‐defective single‐stranded DNA virus, capable of persisting by integrating into the host genome 10, 28. A previous study showed that sustained delivery of anti‐angiogenic factors is essential for successful suppression of tumor growth 29. Direct protein delivery through veins or through intratumoral injection of pharmaceutical reagents has limited efficacy in treating tumors. When given intraventricularly, the short half‐lives of these proteins made them difficult to maintain an appropriate concentration. Frequent intratumoral injections of the therapeutic proteins may elicit local immunological problems and induce certain complications, such as infections and hemorrhage. In a study by Dings, osmotic minipumps were implanted subcutaneously as continuous and systemic modes of treatment for 28 days; however, the long implantation time of such continuous indwelling infusion devices increases the risk of infection, and this approach is often considered too expensive for widespread application 12. In our study, an AAV‐mediated gene transfer technique was used to transduce the therapeutic gene rather than delivering the peptide itself, resulted in high transduction efficiency and a continuous transgene production. In our case, at the end of the eighth week of treatment, the fluorescent light in the tumor cells was still strong in the rAAV‐eGFP group, indicating persistent expression, and the transgene product could be detected for at least 8 weeks in our animal study, indicating the prolonged functional stage of the targeting transgene.

In our study, we first investigate whether the anginex alone had effect on the growth of osteosarcoma, the results of tumor volume and ICH of the anginex group revealed anginex had anti‐angiogenic effect on this tumor, the main mechanism is its inhibition on the proliferation of ECs, and this conclusion is in accordance with our expectation, which formed the basis of the combination therapy. Then, we found the tumor volume of combination treatment group was smaller than the anginex group, and the possible reason was as the following: (1) the radiation of the combination treatment group had effect on ECs; (2) the radiation of the combination treatment group had effect on G‐292 cells, as in the MTT assay the radiation had limited effect on the tumor cells (Fig. 1D), which means this tumor cell is not so sensitive to radiation at this radiation equivalent, and combined with the results of the clone formation assay, then we can conclude that the radiation in combination treatment group may play a role on ECs, not tumor cells. And the decrease in MVD in the radiotherapy group also confirmed this conclusion (Fig. 5A). So what is the mechanism of the combination therapy on ECs? Through the previous researches, we could know that there are mainly two mechanisms of the combined radiation with anti‐angiogenic agents 30, 31, 32. One is so‐called vascular normalization, which means the anti‐angiogenic agents can make the tumor vessels become normal. As we know the vessels of tumor have different morphology with that in other tissues, these vessels are twisted, distorted, and arranged in disorder, which make the tumor cannot get enough oxygen and then become resistant to the chemotherapy and radiotherapy. But the use of anti‐angiogenic agents can reverse these abnormal vessels, then the varied vessels can provide more oxygen to the tumor which let them more sensitive to radiation and also the varied vessels can bring more chemotherapeutic agents to the target cells. The other theory is called “radio‐sensitizing effect” of anginex on tumor vessels. This effect was first described by Dings 12, who used ovarian tumor cells and models to investigate the involved mechanisms and found that anginex functioned as an EC‐specific radiosensitizer because anginex did not affect the in vitro radiosensitivity of MA148 ovarian carcinoma cells, but significantly enhanced the in vitro radiosensitivity of ECs. In our study, as shown in Figure 1D, G‐292 osteosarcoma cells were only weakly radiosensitive at default radiation equivalent, and the following clone formation assay showed the SER_D0_ of Ecs was 1.36, so we tend to ascribe the combination effect to the second theory “radio‐sensitizing effect,” which means the anginex made the ECs become more sensitive to the radiation. Our data suggest that anginex combined with radiation may be an excellent therapeutic strategy for prolonged and effective inhibition of angiogenesis during osteosarcoma development and progression.

Our results also confirmed the inhibitory effects of anginex combined with radiation on pulmonary metastasis. Compared with the combination group in which only one or two small metastatic nodules were observed, the metastatic nodules in the control group mice were large and adhered to the pericardium. Our data revealed that nearly all the mice in the treatment group had pulmonary nodules, which indicated that the AAV‐anginex had no effect on inhibiting early metastasis of the tumor, consistent with the theory that the growth of solid tumors greater than 1–2 mm^3^ is critically dependent on angiogenesis. However, after the metastatic nodules grew to larger than 2 mm^3^, when the vessels became the main supply of nutrition for the tumor, the combination therapy showed good efficacy.

In summary, data from this study suggested that combination regimens of anti‐angiogenic approaches and radiotherapy effectively and synergistically halted the primary tumor growth compared with the individual treatment and control groups, may provide a potential therapeutic avenue for osteosarcoma. However, this strategy did not completely recess the growth and progression of the tumor, suggesting that angiogenesis is a complex mechanism involving multiple factors and pathways. Further investigations are warranted to explore the detailed mechanisms and potential applications of this combination strategy, such as the detailed cellular pathway.

## Conflict of Interest

None declared.

## References

[1] Rosen, G. , S. Suwansirikul , C. Kwon , C. Tan , S. J. Wu , E. J. Beattie , et al. 1974 High‐dose methotrexate with citrovorum factor rescue and adriamycin in childhood osteogenic sarcoma. Cancer 33:1151–1163. 4544836 10.1002/1097-0142(197404)33:4<1151::aid-cncr2820330439>3.0.co;2-8

[2] Renard, A. J. , R. P. Veth , H. W. Schreuder , M. Pruszczynski , J. P. Bökkerink , Q. G. Van Hoesel , et al. 1999 Osteosarcoma: oncologic and functional results. A single institutional report covering 22 years. J. Surg. Oncol. 72:124–129. 10562357 10.1002/(sici)1096-9098(199911)72:3<124::aid-jso3>3.0.co;2-g

[3] Bacci, G. , S. Ferrari , F. Bertoni , P. Ruggieri , P. Picci , A. Longhi , et al. 2000 Long‐term outcome for patients with nonmetastatic osteosarcoma of the extremity treated at the istituto ortopedico rizzoli according to the istituto ortopedico rizzoli/osteosarcoma‐2 protocol: an updated report. J. Clin. Oncol. 18:4016–4027. 11118462 10.1200/JCO.2000.18.24.4016

[4] Ek, E. T. , C. R. Dass , K. G. Contreras , and P. F. Choong . 2007 Pigment epithelium‐derived factor overexpression inhibits orthotopic osteosarcoma growth, angiogenesis and metastasis. Cancer Gene Ther. 14:616–626. 17479108 10.1038/sj.cgt.7701044

[5] Yin, D. , T. Jia , W. Gong , H. Yu , P. H. Wooley , M. P. Mott , et al. 2008 VEGF blockade decelerates the growth of a murine experimental osteosarcoma. Int. J. Oncol. 33:253–259. 18636145

[6] Fu, X. H. , J. Li , Y. Zou , Y. R. Hong , Z. X. Fu , J. J. Huang , et al. 2011 Endostar enhances the antineoplastic effects of combretastatin A4 phosphate in an osteosarcoma xenograft. Cancer Lett. 312:109–116. 21893381 10.1016/j.canlet.2011.08.008

[7] van der Schaft, D. W. , R. P. Dings , Q. G. de Lussanet , L. I. van Eijk , A. W. Nap , R. G. Beets‐Tan , et al. 2002 The designer anti‐angiogenic peptide anginex targets tumor endothelial cells and inhibits tumor growth in animal models. FASEB J. 16:1991–1993. 12397082 10.1096/fj.02-0509fje

[8] Griffioen, A. W. , D. W. van der Schaft , A. F. Barendsz‐Janson , A. Cox , H. S. Boudier , H. F. Hillen , et al. 2001 Anginex, a designed peptide that inhibits angiogenesis. Biochem. J. 354:233–242. 11171099 10.1042/0264-6021:3540233 PMC1221648

[9] Brandwijk, R. J. , R. P. Dings , E. van der Linden , K. H. Mayo , V. L. Thijssen , and A. W. Griffioen . 2006 Anti‐angiogenesis and anti‐tumor activity of recombinant anginex. Biochem. Biophys. Res. Commun. 349:1073–1078. 16970922 10.1016/j.bbrc.2006.08.154

[10] Dong, D. F. , E. X. Li , J. B. Wang , Y. Y. Wu , F. Shi , J. J. Guo , et al. 2009 Anti‐angiogenesis and anti‐tumor effects of AdNT4‐anginex. Cancer Lett. 285:218–224. 19540664 10.1016/j.canlet.2009.05.021

[11] Dings, R. P. , E. S. Van Laar , J. Webber , Y. Zhang , R. J. Griffin , S. J. Waters , et al. 2008 Ovarian tumor growth regression using a combination of vascular targeting agents anginex or topomimetic 0118 and the chemotherapeutic irofulven. Cancer Lett. 265:270–280. 18378392 10.1016/j.canlet.2008.02.048 PMC3042303

[12] Dings, R. P. , B. W. Williams , C. W. Song , A. W. Griffioen , K. H. Mayo , and R. J. Griffin . 2005 Anginex synergizes with radiation therapy to inhibit tumor growth by radio‐sensitizing endothelial cells. Int. J. Cancer 115:312–319. 15688384 10.1002/ijc.20850

[13] Jain, R. K. 2005 Normalization of tumor vasculature: an emerging concept in antiangiogenic therapy. Science 307:58–62. 15637262 10.1126/science.1104819

[14] Jain, R. K. 2001 Normalizing tumor vasculature with anti‐angiogenic therapy: a new paradigm for combination therapy. Nat. Med. 7:987–989. 11533692 10.1038/nm0901-987

[15] Brandwijk, R. J. , I. Nesmelova , R. P. Dings , K. H. Mayo , V. L. Thijssen , and A. W. Griffioen . 2005 Cloning an artificial gene encoding angiostatic anginex: from designed peptide to functional recombinant protein. Biochem. Biophys. Res. Commun. 333:1261–1268. 15979575 10.1016/j.bbrc.2005.06.029

[16] Wang, J. B. , M. D. Wang , E. X. Li , and D. F. Dong . 2012 Advances and prospects of anginex as a promising anti‐angiogenesis and anti‐tumor agent. Peptides 38:457–462. 22985857 10.1016/j.peptides.2012.09.007

[17] Yang, S. Y. , H. Yu , J. E. Krygier , P. H. Wooley , and M. P. Mott . 2007 High VEGF with rapid growth and early metastasis in a mouse osteosarcoma model. Sarcoma 2007:95628. 18274612 10.1155/2007/95628 PMC2225469

[18] Dings, R. P. , D. W. van der Schaft , B. Hargittai , J. Haseman , A. W. Griffioen , and K. H. Mayo . 2003 Anti‐tumor activity of the novel angiogenesis inhibitor anginex. Cancer Lett. 194:55–66. 12706859 10.1016/s0304-3835(03)00015-6

[19] Folkman, J. 1972 Anti‐angiogenesis: new concept for therapy of solid tumors. Ann. Surg. 175:409–416. 5077799 10.1097/00000658-197203000-00014 PMC1355186

[20] Folkman, J. 1995 Angiogenesis in cancer, vascular, rheumatoid and other disease. Nat. Med. 1:27–31. 7584949 10.1038/nm0195-27

[21] O'Reilly, M. S. , T. Boehm , Y. Shing , N. Fukai , G. Vasios , W. S. Lane , et al. 1997 Endostatin: an endogenous inhibitor of angiogenesis and tumor growth. Cell 88:277–285. 9008168 10.1016/s0092-8674(00)81848-6

[22] Pike, S. E. , L. Yao , K. D. Jones , B. Cherney , E. Appella , K. Sakaguchi , et al. 1998 Vasostatin, a calreticulin fragment, inhibits angiogenesis and suppresses tumor growth. J. Exp. Med. 188:2349–2356. 9858521 10.1084/jem.188.12.2349 PMC2212424

[23] Tolsma, S. S. , O. V. Volpert , D. J. Good , W. A. Frazier , P. J. Polverini , and N. Bouck . 1993 Peptides derived from two separate domains of the matrix protein thrombospondin‐1 have anti‐angiogenic activity. J. Cell Biol. 122:497–511. 7686555 10.1083/jcb.122.2.497 PMC2119646

[24] O'Reilly, M. S. , L. Holmgren , Y. Shing , C. Chen , R. A. Rosenthal , M. Moses , et al. 1994 Angiostatin: a novel angiogenesis inhibitor that mediates the suppression of metastases by a Lewis lung carcinoma. Cell 79:315–328. 7525077 10.1016/0092-8674(94)90200-3

[25] van der Schaft, D. W. , E. A. Toebes , J. R. Haseman , K. H. Mayo , and A. W. Griffioen . 2000 Bactericidal/permeability‐increasing protein (BPI) inhibits angiogenesis via induction of apoptosis in vascular endothelial cells. Blood 96:176–181. 10891448

[26] Morishita, T. , Y. Mii , Y. Miyauchi , S. Miura , K. Honoki , M. Aoki , et al. 1995 Efficacy of the angiogenesis inhibitor O‐(chloroacetyl‐carbamoyl) fumagillol (AGM‐1470) on osteosarcoma growth and lung metastasis in rats. Jpn. J. Clin. Oncol. 25:25–31. 7745819

[27] Luo, J. , Y. Luo , J. Sun , Y. Zhou , Y. Zhang , and X. Yang . 2015 Adeno‐associated virus‐mediated cancer gene therapy: current status. Cancer Lett. 356:347–356. 25444906 10.1016/j.canlet.2014.10.045 PMC4259838

[28] Flotte, T. R. , S. A. Afione , and P. L. Zeitlin . 1994 Adeno‐associated virus vector gene expression occurs in nondividing cells in the absence of vector DNA integration. Am. J. Respir. Cell Mol. Biol. 11:517–521. 7946381 10.1165/ajrcmb.11.5.7946381

[29] Ma, H. I. , S. Z. Lin , Y. H. Chiang , J. Li , S. L. Chen , Y. P. Tsao , et al. 2002 Intratumoral gene therapy of malignant brain tumor in a rat model with angiostatin delivered by adeno‐associated viral (AAV) vector. Gene Ther. 9:2–11. 11850717 10.1038/sj.gt.3301616

[30] Classen, J. , and W. Budach . 2003 Antiangiogenesis and radiotherapy: what is the role of combined modality treatment? Curr. Med. Chem. Anticancer Agents 3:375–382. 12871084 10.2174/1568011033482305

[31] Jain, R. K. 2014 Antiangiogenesis strategies revisited: from starving tumors to alleviating hypoxia. Cancer Cell 26:605–622. 25517747 10.1016/j.ccell.2014.10.006 PMC4269830

[32] Hsu, H. W. , N. R. Wall , C. T. Hsueh , S. Kim , R. L. Ferris , C. S. Chen , et al. 2014 Combination antiangiogenic therapy and radiation in head and neck cancers. Oral Oncol. 50:19–26. 24269532 10.1016/j.oraloncology.2013.10.003

